# High prevalence of oral potentially malignant disorders and risk factors in a semi-urban brazilian city: a population-based cross-sectional study

**DOI:** 10.4317/medoral.24747

**Published:** 2021-05-23

**Authors:** Allan Vinícius Martins-de-Barros, Ana Maria Ipólito Barros, Caio César Gonçalves Silva, Letícia Francine Silva Ramos, Stefânia Jerônimo Ferreira, Fábio Andrey da Costa Araújo, Emanuel Dias de Oliveira e Silva, Marianne de Vasconcelos Carvalho

**Affiliations:** 1 DDS. Post-Graduation Program in Dentistry / Oral Medicine and Pathology. School of Dentistry, University of Pernambuco. Recife, Brazil; 2 DDS, MsC. Post-Graduation Program in Dentistry / Oral and Maxillofacial Surgery. School of Dentistry, University of Pernambuco. Recife, Brazil; 3 Dentistry student. School of Dentistry, University of Pernambuco. Arcoverde, Brazil; 4 DDS, MSc, PhD. Professor. School of Dentistry, University of Pernambuco. Arcoverde, Brazil; 5 DDS, MSc, PhD. Professor. School of Dentistry, University of Pernambuco. Recife, Brazil

## Abstract

**Background:**

Oral Potentially Malignant Disorders (OPMDs) are defined as lesions with a greater likelihood of progressing to cancer. Population-based studies that evaluate the prevalence of OPMDs are scarce in Brazil. The aim of the present study was to determine the prevalence of OPMDs and associated risk factors in a semi-urban Brazilian population.

**Material and Methods:**

This is a cross-sectional study, whose universe included individuals aged 40 years or older residing in a medium-sized city of northeastern Brazil. Data collection was divided into two steps: interview and oral examination. The outcome variable was the presence of OPMDs. The predictor variables were sociodemographic characteristics and risk habits. The bivariate analysis was performed through chi-square test. The crude prevalence ratios (PR) and its respective 95% confidence intervals (CI) were calculated. Poisson regression analysis with robust variance was used to calculate adjusted PRs and 95% CI.

**Results:**

Three hundred fourteen individuals were included in the study. When asked about risk habits, 58.9% reported being current smokers or ex-smokers and 62.2% reported being current drinkers or ex-drinkers. The prevalence of OPMDs was 7.6% and was significantly higher among individuals with black skin color (*p* < 0.001), alcohol users (*p* = 0.017), and individuals with both tobacco and alcohol habits (*p* = 0.012).

**Conclusions:**

Therefore, the population in the present study had a high frequency of risk habits associated with PMDs of the oral cavity.

** Key words:**Oral mucosal lesions, oral cancer, oral potentially malignant disorders, prevalence.

## Introduction

Oral cancer is considered an important public health problem (1,2). Even with advances in treatment, the five-year survival rate has not increased in decades. Squamous cell carcinoma (SCC) is the most frequent malignant tumor in the oral cavity (3). Oral Potentially Malignant Disorders (OPMDs) are defined as lesions with a greater likelihood of progressing to cancer. In such cases, the tissue exhibits morphological and cellular changes and has a greater tendency to undergo malignant transformation into an SCC compared to normal epithelium (4).

Approximately 95% of cases of oral cancer occur in individuals older than 40 years of age (3), the majority of whom are men, users of tobacco and alcohol, and have a low socioeconomic status (5,6). Knowledge on the prevalence and profile of the distribution of OPMDs in populations at risk is fundamental to the establishment of public prevention and control policies.

Population-based studies that evaluate the prevalence of OPMDs are scarce in Brazil, especially in the northeastern region of the country. Therefore, the aim of the present study was to determine the prevalence of OPMDs and associated risk factors in individuals 40 years of age or older in a population of a medium-sized semi-urban city in the state of Pernambuco, Brazil.

## Material and Methods

- Study design and population

The present study received approval from the institutional review board of the University of Pernambuco (CAAE: 31240020.7.0000.5191) and was conducted in accordance with the guidelines of the Strengthening the Reporting of Observational Studies in Epidemiology (STROBE) statement for cross-sectional studies.

An observational, cross-sectional, prevalence study was conducted through a population-based epidemiological survey in a medium-sized city in the semiarid region of the state of Pernambuco (northeastern Brazil). Study population was composed of male and female individuals aged 40 years or older residing in the area of coverage of a primary care unit. The exclusion criteria were cognitive, neurological, or psychiatric disorders that impeded answering the questionnaire, the non-localization of individuals at their homes after three attempts at different times of the day, and refusal to participate in any of the steps of the study.

- Sample size calculation and sampling procedure

The sample size was calculated using the highest prevalence for OPMDs (10.54%) reported in a systematic review conducted by Mello *et al*. (7) Considering a 99% confidence interval (α = 0.01), 5% accepTable margin of error, a design effect of 1.0, and adjustment for a possible 25% non-response rate, the sample was defined as 314 individuals.

Individuals belonging to the target population (units of analysis) and their respective residential addresses were identified and listed based on e-SUS AB records (electronic primary care records of the Brazilian public healthcare system) kindly granted by the Municipal Secretary of Health. A simple random sampling procedure was employed with the aid of a computer-generated random number Table (available at www.random.org) to select the sample.

- Data collection in field

The data collection instrument was a questionnaire with closed-ended questions addressing sociodemographic characteristics (sex, age, and self-reported skin color), risk habits (tobacco and alcohol use), and the presence of oral lesions suspected of being cancer or OPMDs.

Data collection was performed in the respondents’ homes by three duly trained researchers who had undergone a calibration exercise for the diagnosis of oral lesions. The collection procedure was divided into two steps: interview and oral examination. The interview consisted of the administration of the questionnaire. The soft tissues of the oral cavity were examined with the aid of an artificial light and mouth mirror. The examination was performed systematically on all anatomic structures of the mouth and oropharynx through a visual inspection and palpation. The researcher recorded the presence/absence of lesions of the oral mucosa exhibiting clinical characteristics consistent with oral cancer, leukoplakia, or erythroplakia based on the criteria established by Warnakulasuriya *et al*. (8) and noted the clinical characterization of the lesions.

- Database and statistical analysis

The database of the study was created with the aid of the Statistical Package for Social Sciences (SPSS® version 20.0.0). For the bivariate analysis, the variables were categorized dichotomously. The outcome variable was the presence of OPMDs. The predictor variables were sociodemographic characteristics and risk habits. The bivariate analysis was performed through chi-square test. The crude prevalence ratios (PR) and its respective 95% confidence intervals (CI) were calculated. Poisson regression analysis with robust variance was performed for the calculation of adjusted PRs with 95% CI. Variables with a *p-value* < 0.20 in the bivariate analysis were incorporated into the multivariate model and those with a *p-value* < 0.05 after the adjustments were considered significantly associated with the outcome.

## Results

**Figure 1:**
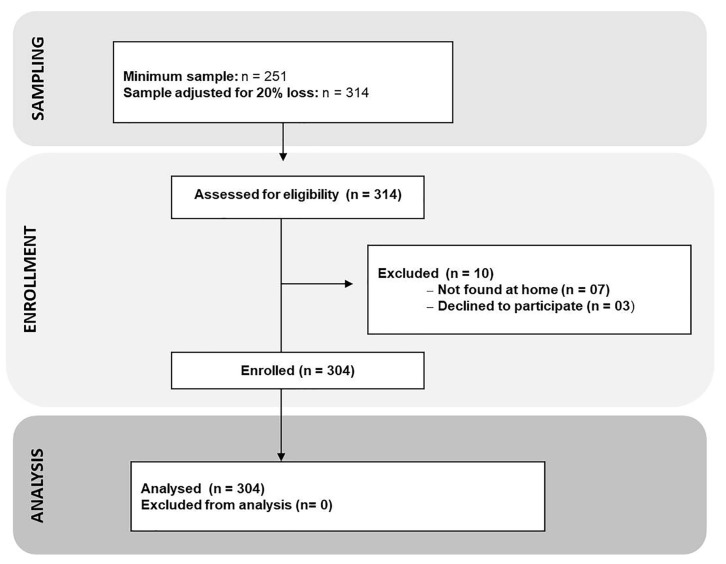
Participant Flowchart.

Three hundred fourteen individuals were selected to compose the sample of the present study. Seven were not found at their homes after three attempts and three declined to participate in the study. Thus, 304 individuals completed all steps of the data collection and composed the final sample (Fig. 1). The sociodemographic characteristics of the participants are displayed in Table 1. Age ranged from 40 to 94 years (mean: 62.33 ± 13.57 years). Women (69.4%) and individuals with brown skin color (62.2%) accounted for the majority of the sample.

When asked about risk habits, 58.9% reported being current smokers or ex-smokers and 62.2% reported being current drinkers or ex-drinkers. Only 62 individuals (20.4%) never used tobacco or alcoholic beverages. Among the smokers and ex-smokers, the largest portion (49.2%) reported smoking less than 10 cigarettes/day and 7.8% reported smoking more than 40 cigarettes/day. The type of cigarette most frequently used was of paper (86%). Regarding the alcohol intake pattern, 97 individuals reported the use of more than one type of beverage. Beer was the most frequent (69.9% of drinkers), whereas 27 of those who drank reported drinking beer, wine, cachaça (distilled spirit made from fermented sugarcane juice), and other distilled spirits. The quantity of drinks per day that each individual consumed was also investigated: 63% reported occasional use and 14.3% reported having or having had more than six drinks per day. Total of 47.8% of ex-smokers and 41.8% of ex-drinkers reported having quit the habit more than 20 years earlier. The absolute and relative frequencies of smoking and drinking patterns are displayed in Table 1.

Smoking experience (smokers and ex-smokers) was significantly associated with skin color (*p* = 0.002). Alcohol experience (drinkers and ex-drinkers) was significantly associated with both age (*p* < 0.001) and sex (*p* < 0.001) (Table 2).

The physical examinations of the oral cavity revealed lesions suspected of being OPMDs in 23 individuals (7.6%). Seventeen lesions were characterized as leukoplakia, five were characterized as erythroplakia, and only one was characterized as erythroleukoplakia. No lesions suggestive of SCC were identified.

The crude PRs for OPMDs and *p-value*s from the chi-squared test are displayed in Table 3. In the bivariate analysis, the presence of OPMDs was significantly associated with skin color (*p* = 0.001) and alcohol use (*p* = 0.047).

The adjusted PRs and respective 95% CIs are displayed in Table 4. The prevalence of OPMDs was significantly higher among individuals with black skin color (PR: 4.783; 95% CI: 2.327 to 9.831; *p* < 0.001), alcohol users (PR: 3.207; 95% CI: 1.229 to 8.369; *p* = 0.017), and individuals with both tobacco and alcohol habits (PR: 4.143; 95% CI: 1.369 to 12.541; *p* = 0.012).

**Table 1:**
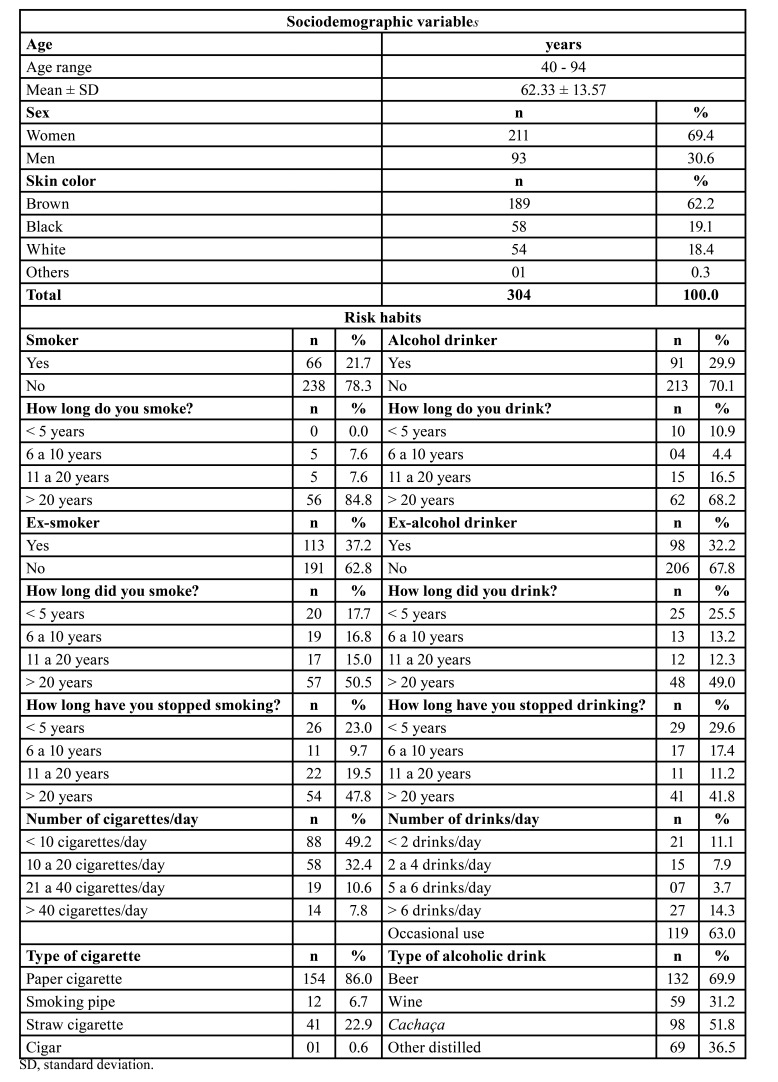
Sociodemographic charactesitics and frequency of risk habits among the participants of the study.

**Table 2:**
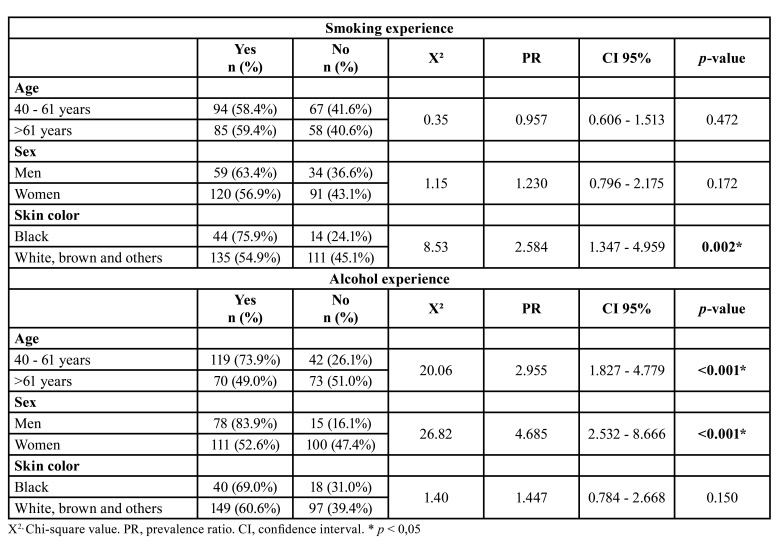
Relationship between smoking and alcohol experience and sociodemographic characteristics among the participants of the study.

**Table 3:**
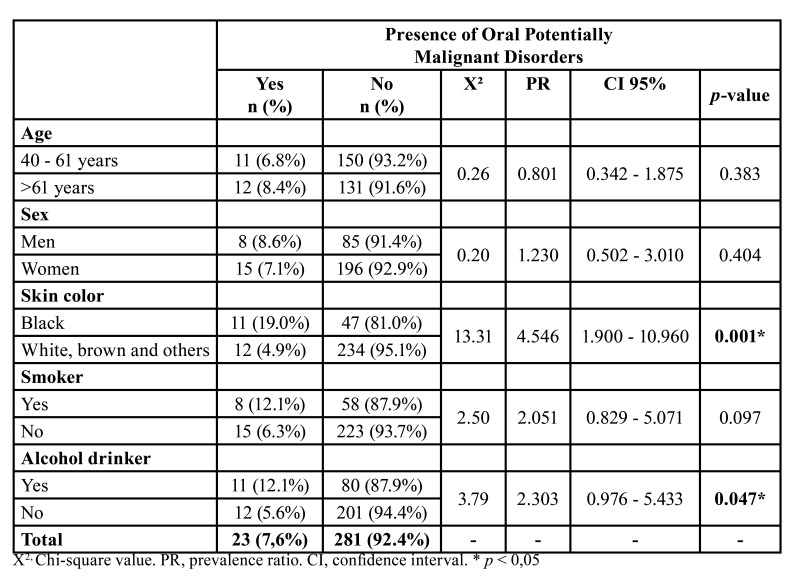
Relationship between Oral Potentially Malignant Disorders and sociodemographic characteristics and risk habits among the participants of the study.

**Table 4:**
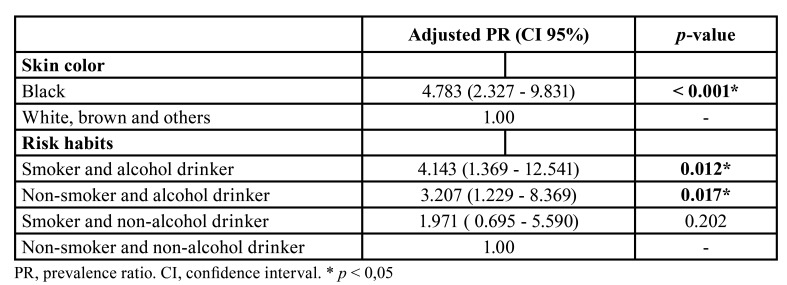
Adjusted Prevalence Ratios and its respective 95% Confidence Interval for the association between the presence of Oral Potentially Malignant Disorders and skin color, smoking and alcohol drinking habits.

## Discussion

SCCs and OPMDs are pathological changes originating in the lining tissue of the oral cavity and are directly associated with demographic characteristics, such as age, as well as risk habits, such as smoking and alcohol intake (9). As the prevalence of these lesions and associated risk factors varies significantly in different populations and geographic regions, knowledge on the local-regional epidemiological status is fundamental to the establishment of prevention measures and the early diagnosis of these conditions.

The World Health Organization strongly recommends population-based epidemiological surveys to determine the prevalence of oral lesions related to cancer (10). However, few studies with this methodological design are found in the scientific literature. In Brazil, prevalence data regarding malignant and potentially malignant lesions of the oral cavity are mainly from studies conducted at institutions or healthcare establishments with non-random samples, which substantially increases the risk of bias and compromises the external validity of the findings.

The pooled global prevalence of OPMDs calculated in a meta-analysis of a systematic review conducted by Mello *et al*. (7) was 4.47%. When only considering leukoplakia and erythroplakia, the prevalence was 4.28% (9). In the present study, a much higher rate of PMDs in the oral cavity clinically characterized as leukoplakia, erythroplakia, and erythroleukoplakia was found in the population studied (7.6%). Population-based cross-sectional studies conducted in Brazil, such as the investigation by Ferreira *et al*. (10), report a much lower prevalence of oral leukoplakia (2.3%) and erythroplakias (0.3%) in rural workers. In contrast, Pivovar *et al*. (11) found a nearly 25% rate of leukoplakia in a population classified as having a high risk of oral cancer. The considerable variability in the prevalence of these disorders may be related to sociodemographic characteristics and the frequency of cultural practices considered risk habits in different populations.

Smoking and alcohol use are well-established etiological factors for OPMDs and play an equally important role in the progression of these lesions (9,12). In the multivariate model of the present study, the prevalence of OPMDs was significantly higher among individuals who used tobacco or alcoholic beverages. Moreover, due to the synergistic effect, the prevalence was 4.14-fold higher among those who reported the concomitant use of these two substances.

Although the prevalence of these habits has diminished in Brazil in recent years, there is a marked inequality in the reach and effectiveness of prevention measures in different socioeconomic strata (13,14,15). Populations who live in the interior of the state are expected to behave differently compared to those who live in large metropolises in terms of risk factors associated with OPMDs and oral cancer. The rates of smoking and alcohol use in the present study are higher than the average for Brazil as a whole and for the northeastern region in particular (13,14). This finding is compatible to data reported by Amarasinghe *et al*. (16) for a rural population in the interior or Sri Lanka. Thus, regional differences in the prevalence of OPMDs seem to accompany those of tobacco and alcohol use.

There is no consensus in the literature on the association between sex and oral OPMDs, as the distribution of such disorders is directly influenced by socioeconomic and cultural aspects (7). Although some clinical types of OPMDs, such as actinic cheilitis, are more prevalent among men (10), no significant difference between the sexes was found regarding the prevalence of OPMDs in the present study. This may partially be explained by the greater similarity in the social behavioral patterns of men and women in instate populations regarding risk habits, especially smoking. Indeed, no significant difference in smoking experience was found between the sexes.

Few studies have analyzed the distribution of OPMDs in different ethnic groups. In a systematic review conducted by Mello *et al*. (7), the authors found a greater frequency of OPMDs in “light skin” individuals. In contrast, the prevalence was 4.78-fold greater among black skin individuals in the present investigation. This difference is probably not due to biological factors, as the quantity and distribution of melanin in tissues is neither a risk factor nor protection factor for the development of OPMDs, mainly because these are lesions for which ultraviolet solar radiation is not a determinant etiological factor. The greater prevalence in black individuals seems to be related to the marked social inequality and greater difficulty in gaining access to services and information. Kravietz *et al*. (17) highlighted important racial inequality regarding access to information and screening services for head and neck cancer in the United States. Such inequality tends to be greater in municipalities distant from metropolises, which contributes to an increased risk of oral cancer and OPMDs in these populations.

Although lesions located in the mouth are accessible to a visual inspection and palpation (18), the diagnosis is generally performed when such lesions are already in late stages (3). Although there is little current evidence on the effectiveness of screening programs for oral cancer (19), early detection of OPMDs in stages prior to a possible malignant transformation has the potential to reduce morbidity and mortality rates related to these conditions, especially in individuals exposed to risk factors (7,18). However, there is a lack of decentralized programs for the early identification of oral lesions in instate regions (10). In this regard, the data of the present study can assist in guiding such programs to populations at greater risk.

The present study has limitations that should be considered. The cross-sectional design does not enable the establishment of a cause-and-effect relationship between exposure and outcome in the analysis of associations between variables. The lack of histopathological data impedes a more in-depth analysis of morphological changes and the potential for the progression to malignancy in the lesions clinically diagnosed as OPMDs. Moreover, the lack of methodological standardization among prevalence studies in the literature regarding sampling procedures and the diagnosis of lesions compromises the uniformity of the data and hinders the comparison of the results.

In conclusion, the population in the present study had a high prevalence of OPMDs and associated risk habits. The majority of lesions were clinically characterized as leukoplakia. The prevalence of OPMDs was higher among individuals with black skin color and those who had the concomitant habits of smoking and alcohol use. These findings underscore the importance of intensifying policies aimed at primary prevention and the early diagnosis of OPMDs and oral cancer with a focus on the dissemination of information and the control of risk habits, especially in more vulnerable geographic areas and socioeconomic strata, as lifestyle and living conditions in rural and semi-urban areas differ considerably from those found in large urban centers.
